# TRP channels: a provocative rationalization for local Ca^2+^ control in arterial tone development

**DOI:** 10.3389/fphys.2024.1374730

**Published:** 2024-02-28

**Authors:** Mohammed A. El-Lakany, Donald G. Welsh

**Affiliations:** ^1^ Department of Physiology & Pharmacology, Robarts Research Institute, Schulich School of Medicine, University of Western Ontario, London, ON, Canada; ^2^ Department of Pharmacology and Toxicology, Faculty of Pharmacy, Alexandria University, Alexandria, Egypt

**Keywords:** TRP channels, Ca^2+^ waves, pharmacomechanical coupling, receptor operated Ca^2+^ channels, vascular ion channels

## Abstract

Arterial networks are controlled by the consolidated output of stimuli that set “how much” (magnitude) and “where” (distribution) blood flow is delivered. While notable changes in magnitude are tied to network wide responses, altered distribution often arises from focal changes in tone, whose mechanistic foundation remains unclear. We propose herein a framework of focal vasomotor contractility being controlled by pharmacomechanical coupling and the generation of Ca^2+^ waves via the sarcoplasmic reticulum. We argue the latter is sustained by receptor operated, transient receptor potential (TRP) channels through direct extracellular Ca^2+^ influx or indirect Na^+^ influx, reversing the Na^+^/Ca^2+^ exchanger. We view this focal regulatory mechanism as complementary, but not redundant with, electromechanical coupling in the precision tuning of blood flow delivery.

## 1 Introduction

Arterial networks are rationalized as an intricate system of segments which serves to set blood flow magnitude and adapt its distribution dependent on local energetic demands. Arteries are described as single-layered tubes of endothelial cells surrounded by smooth muscle cells arranged in a circular fashion. Endothelial cells are arranged parallel to the axis of blood flow and as they are in intimate contact with blood, they respond to mechanical forces like shear stress. They are strongly coupled to one another, unlike smooth muscle, and thus constitute the primary pathway for electrical and chemical signals to spread along vasculature (Mironova et al., 2024). Smooth muscle cells are circumferentially arranged and retain the contractile machinery responsible for altering arterial tone. Tone within arterial networks is governed by multiple stimuli, the most important being intravascular pressure, blood flow, neural activity, and a range of endothelial derived factors. Alterations in smooth muscle [Ca^2+^] are the principal driver of arterial tone, with cytosolic levels largely set by the activity of voltage gated Ca^2+^ channels. The standard mechanistic paradigm, commonly termed electromechanical coupling, is straightforward in thought, thousands of smooth muscle cells exposed to a defined stimulus (e.g., intravascular pressure) subtly change their conductance to Na^+^, Cl^−^ or K^+^. With the aid of gap junction, charge spreads among coupled vascular cells, producing a homogenous membrane potential response that gates L- and to a lesser extent T-type Ca^2+^ channels. The ensuing cytosolic Ca^2+^ response modulates myosin light chain kinase and consequently the contractile state of smooth muscle. These integrated responses have garnered deep interrogation as they set the foundation of blood flow control and coordinate the dilations that increase tissue perfusion with rising metabolic demand. What is often overlooked in such examinations is the need to discretely tune blood flow distribution by allowing segments, in particular terminal/pre-capillary arterioles, to focally constrict independent of the broader structure (Sweeney et al., 2016; Grubb et al., 2020; Zambach et al., 2021). Such behavior is observable in isolated vessels and *in-vivo* to focal agent delivery, and it is insensitive to membrane potential and the influx of Ca^2+^ via L-type Ca^2+^ channels. Voltage insensitive contraction is often termed pharmacomechanical coupling, and its transduction is tied to signaling pathways activated G-protein coupled receptors.

**FIGURE 1:**
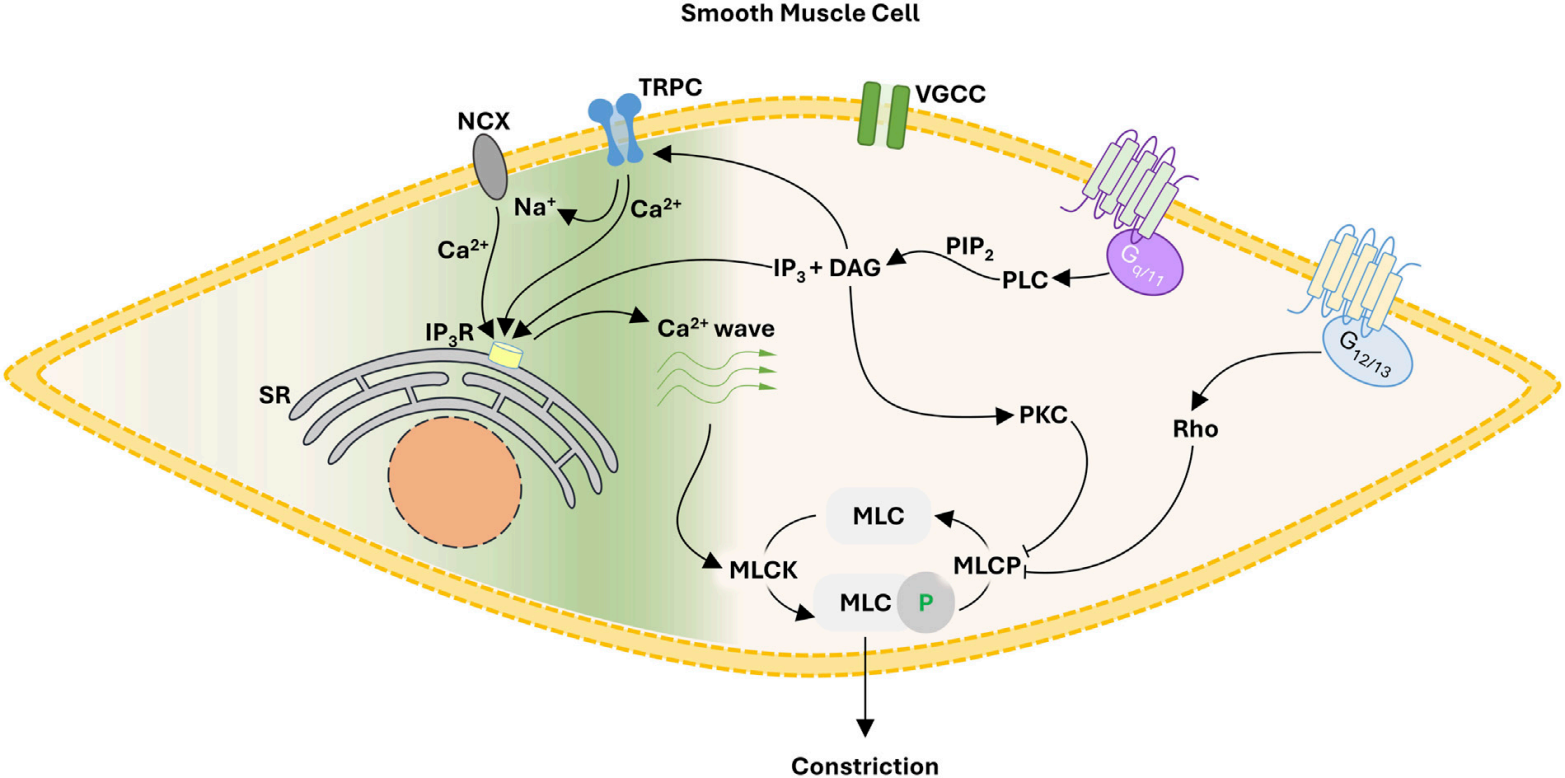
Proposed framework for focal constriction through pharmacomechanical coupling, a process independent of membrane potential and VGCC. A G_q/11_ signaling pathway inhibits MLCP while activating TRPC cationic channels. The latter, operating as a Ca^2+^ influx pathway or by reversing NCX transport, facilitates the generation of Ca^2+^ waves necessary for MLCK activation. Abbreviations: NCX, Na^+^/Ca^2+^ exchanger; TRPC, canonical transient receptor potential channel; VGCC, voltage-gated Ca^2+^ channel; SR, sarcoplasmic reticulum; IP_3_, inositol triphosphate; DAG, diacyl glycerol; PLC, phospholipase C; PIP_2_, phosphatidylinositol 4,5-bisphosphate; MLC, myosin light chain; MLCK, MLC kinase; MLCP, MLC phosphatase.

The query addressed herein centers on how focal constrictor events can be generated independent of electromechanical coupling. Reason dictates careful consideration of pharmacomechanical coupling and the regulation of myosin light chain phosphatase (MLCP) through G_q/11_ and G_12/13_ coupled signal pathways (Figure 1). G_q/11_ coupled receptors (e.g., α_1_ adrenoreceptors) activate phospholipase C-β, the ensuing hydrolysis of phosphatidylinositol 4,5-bisphosphate leading to diacylglycerol and inositol triphosphate (IP_3_) production. The former leads to the activation of protein kinase C and the phosphorylation of CPI-17, an upstream inhibitor of the MLCP catalytic subunit (PP1c). In contrast, G_12/13_ coupled receptors (e.g., thromboxane A_2_ receptor) activate the monomeric G-protein RhoA and then Rho-kinase to regulate the MLCP targeting subunit, MYPT1 via two key phosphorylation sites (T-697 & T-855). While MLCP inhibition is logically essential for focal vasoconstrictor control, a Ca^2+^ signal, one modest in magnitude and uncoupled to voltage, is still required for myosin light chain kinase (MLCK) activation. While the mechanistic foundation of this Ca^2+^ signal remains unsettled, it is intriguing to consider Ca^2+^ waves which originate from the sarcoplasmic reticulum (SR) and are triggered by IP_3_ production and extracellular Ca^2+^ influx through an elusive Ca^2+^ permeable pore. The subsequent sections will consider the molecular identity of that Ca^2+^ permeable pore and how a voltage-insensitive Ca^2+^ source shapes focal tone development and blood flow distribution throughout integrated arterial networks.

## 2 The sarcoplasmic reticulum and Ca^2+^ waves

Vascular smooth muscle retains sarcoplasmic reticulum, an internal store that releases Ca^2+^ upon activation of ryanodine or IP_3_-sensitive receptors (IP_3_R). Ca^2+^ release takes the form of several definable events, the two most common being “Ca^2+^ sparks” and “Ca^2+^ waves.” Ca^2+^ sparks, as discerned by Nelson et al. (1995) are focal voltage-dependent events that rapidly activate large-conductance Ca^2+^-activated K^+^ channels, thus initiate spontaneous transient outward currents (STOCs). In resistance arteries, STOCs drive a feedback hyperpolarization that moderates constriction initiated by stimuli like intravascular pressure. Ca^2+^ waves are slower events (1–3 s, duration) characterized by a wave front spreading from end-to-end within one cell and then asynchronously among neighboring cells. Their spatial/temporal heterogeneity is notably distinct from the synchronous, oscillatory behavior underpinning arterial vasomotion (Seppey et al., 2009). Asynchronous Ca^2+^ waves display voltage-insensitive properties and their abolishment moderates tone development to superfused agonist (Jaggar and Nelson, 2000; Mufti et al., 2010). Ca^2+^ waves reflect regenerative Ca^2+^-induced Ca^2+^-release among neighboring IP_3_ and/or ryanodine receptors, an event dependent on IP_3_ production and extracellular Ca^2+^ influx. The latter would be presumptively important to driving the initial opening of IP_3_Rs or to refilling of the SR. What is the most likely identity of this Ca^2+^ permeable pore?

## 3 Ca^2+^ permeable pores and Ca^2+^ waves

### 3.1 The fabled “receptor operated Ca^2+^ channel”

Receptor operated Ca^2+^ channels were first rationalized by Somlyo and Somlyo (1968) to partly explain persistent agonist-induced tone following L-type Ca^2+^ channel blockade. Despite this early conceptualization, the identity of “receptor operated Ca^2+^ channels” remained elusive for decades as its electrophysiological and pharmacological fingerprint was variable and difficult to define. A *bona fide* target emerged in the 21st century, with the isolation of transient receptor potential (TRP) channels, named after a mutant *drosophila* strain with visual cue blindness (Cosens and Manning, 1969). Molecular studies revealed TRP channels as a diverse group of cation permeable pores, comprised of 4 pore forming subunits, 6 transmembrane domains each and intracellular termini whose molecular diversity imparts unique regulatory properties (Hellmich and Gaudet, 2014). There are six subfamilies, the first identified being the canonical (TRPC) class (Wes et al., 1995) followed by those: 1) modulated by “vanilloid”-like molecules (TRPV; Caterina et al., 1997); and 2) retaining C-terminal repeats related to the “ankyrin” protein (TRPA; Jaquemar et al., 1999). Genomic analysis of disease pathologies subsequently identified the final three subfamilies that being the melanoma metastasis/TRP melastatin (TRPM; Duncan et al., 1998), the autosomal dominant polycystic kidney disease/TRP polycycstin (TRPP; Mochizuki et al., 1996), and the human mucolipidosis type IV/TRP mucolipin (TRPML; Bargal et al., 2000). While multiple TRP subunits are expressed in vascular smooth muscle, the canonical subclass has garnered particular attention as activation is coupled to G_q_ signal transduction, rendering it “receptor operated.” TRPC channels pass both mono- (e.g., Na^2+^) and di- (Ca^2+^) valents, their relative permeability set by subunit composition (Bolton, 1979; Putney and Tomita, 2012). As such, they can operate as a depolarizing current and/or a Ca^2+^ influx pathway that drives: 1) electrical feedback, via the large conductance Ca^2+^ activated K^+^ channel (Earley et al., 2005); or 2) Ca^2+^ wave generation (Figure 1). We will consider the latter below, with attention focused on two TRPC channel subunits for which foundational data is available. We acknowledge *a priori* TRPC channel subunits do heteromultimerize, but due to a lack of functional observations, discussion on this topic cannot be advanced.

#### 3.1.1 TRPC6

TRPC6 is ubiquitously expressed in vascular smooth muscle and recognized for its relatively high permeability to Ca^2+^ over Na^+^ (5-6:1; Hofmann et al., 1999; Dietrich et al., 2003). When expressed in HEK293 cells, TRPC6 channels displayed two prominent properties, that being dual rectification and the induction of a cytosolic Ca^2+^ response following activation (Dietrich et al., 2003; Estacion et al., 2006). TRPC6 were first reported as a G_q_-coupled, receptor operated Ca^2+^ channel in fibroblast-like monkey cells (Boulay et al., 1997), an observation later transposed into vascular smooth muscle (Inoue et al., 2001; Anfinogenova et al., 2011). As to the latter, Ca^2+^ influx through TRPC6 channels is under the regulatory control of catecholamines (Inoue et al., 2001) along with vasopressin (Maruyama et al., 2006) and angiotensin II (Saleh et al., 2006). Evidence that Ca^2+^ influx via TRPC6 might facilitate to SR Ca^2+^ wave generation is developing but somewhat indirect, with evidence showing that: 1) channel clusters form in plasma membrane adjacent to the SR; and 2) TRPC6 knockdown is associated with a reduction in agonist induced, Ca^2+^ oscillations in cultured smooth muscle cells (Li et al., 2008).

#### 3.1.2 TRPC3

TRPC3 cation channels shares similarities to TPRC6 being activated by G_q_-coupled agonists (Liu et al., 2009) and displaying inward and outward rectification (Kamouchi et al., 1999). It is however distinct from TRPC6, in that basal activity is higher (Dietrich and Gudermann, 2007) and selectivity to Ca^2+^ over Na^+^ is reduced (1.6:1; Kamouchi et al., 1999). Arteries from TRPC3^−/−^ mice display diminished constriction to phenylephrine (Yeon et al., 2014), presumptively due to TRPC3 inducing a depolarization that triggers L-type Ca^2+^ channels (Reading et al., 2005; Pereira da Silva et al., 2022). While reasoned, more recent work has suggested that TRPC3 may also act as a receptor operated Ca^2+^ channel, enabling tone development through Ca^2+^ waves induction. Of particular interest are findings showing that TRPC3 binds to IP_3_R (Yuan et al., 2003; Adebiyi et al., 2012) through its calmodulin/IP_3_R binding domain (Zhang et al., 2001). This C-terminus motif impacts channel gating (Wedel et al., 2003) and presumptively the concomitant Ca^2+^ influx needed to initiate or sustain SR Ca^2+^ waves generation (Curcic et al., 2022). Note, TRPC1 also been alluded to interact with IP_3_Rs although findings remain limited (Yuan et al., 2003).

#### 3.1.3 Indirect role for TRPC channels?

While it reasoned to suggest that Ca^2+^ influx through TRPC channels induces SR Ca^2+^ waves directly, there is a body of literature which suggests their role in internal Ca^2+^ mobilization is indirect. Consider the Na^+^/Ca^2+^ exchanger, an antiporter that employs the Na^+^ electrochemical gradient to facilitate the efflux of Ca^2+^. Due to its electrogenic nature, the Na^+^/Ca^2+^ exchanger can operate in reverse mode, enabling the Ca^2+^ influx presumptively needed to drive Ca^2+^ wave generation (Blaustein and Lederer, 1999) (Figure 1). This perspective aligns with past work showing that Na^+^/Ca^2+^ exchanger inhibition not only diminishes Ca^2+^ waves but the generation of nifedipine-insensitive tone (Dai et al., 2006; Syyong et al., 2009). Antiporter reversal occurs when cells are depolarized or when local intercellular [Na^+^] rises, presumptively due to TRPC channel activation and monovalent influx (Rosker et al., 2004; Lemos et al., 2007). While evidence is somewhat circumspect, past studies have shown a physical association between TRPC channels and NCX. TRPC3 coimmunoprecipitates with NCX in transfected HEK cells (Rosker et al., 2004), while in vascular smooth muscle, Na^+^/Ca^2+^ exchangers are thought to cluster in association with TRPC6 adjacent to the sarcoplasmic reticulum (Poburko et al., 2007).

## 4 Focal constriction and the spatial throttling of blood supply

**FIGURE 2:**
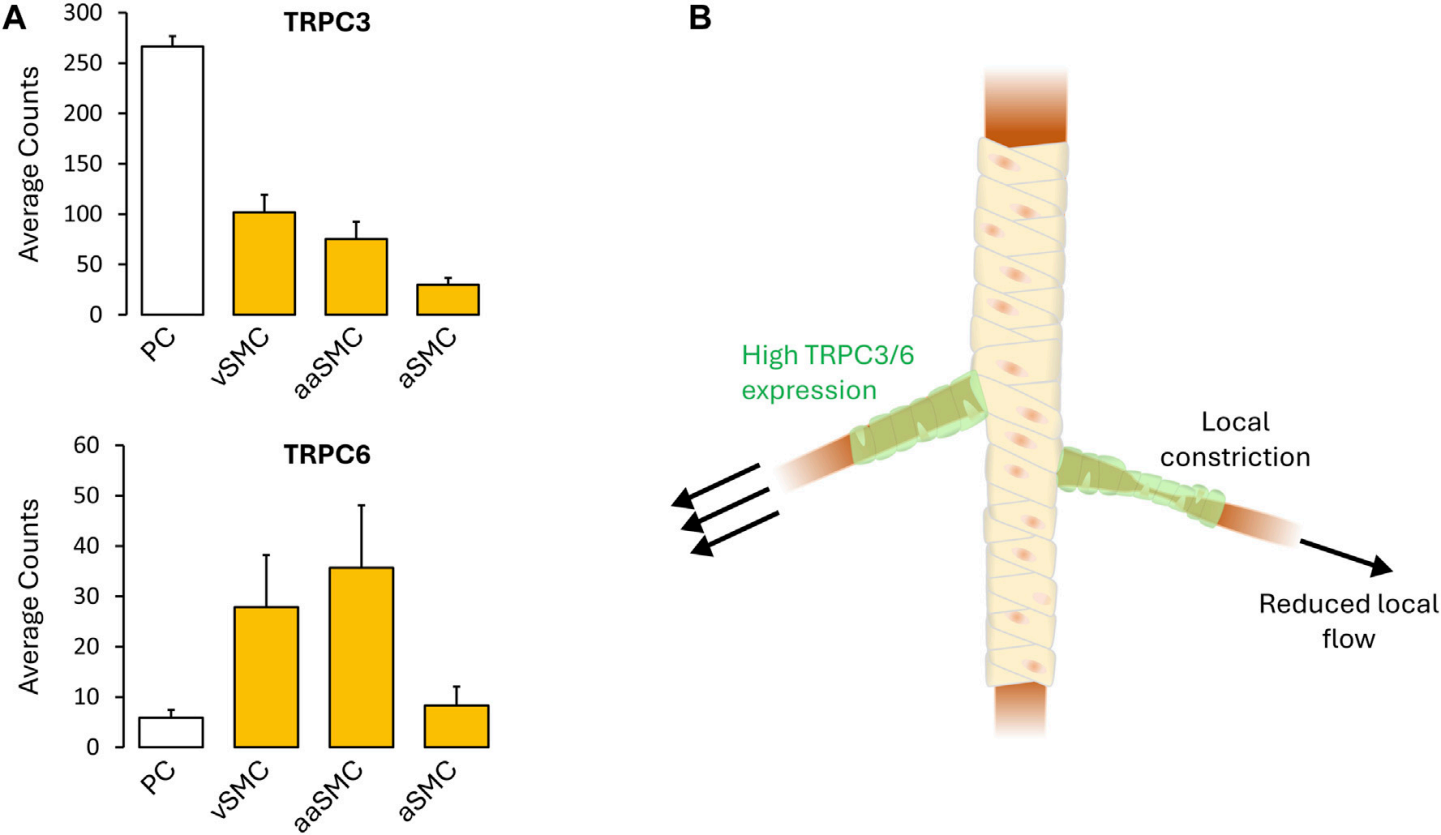
**(A)** TRPC3/6 expression (defined as cellular transcript counts/cell) in the mouse brain mural cells, as determined by single-cell RNA sequencing. Note the strong TRPC3/6 expression. Figures provided by http://betsholtzlab.org/VascularSingleCells/database.html (He et al., 2018; Vanlandewijck et al., 2018). **(B)** Spatial throttling of blood supply through pharmacomechanical coupling independent of an integrated electrical signal. Abbreviations: PC, pericytes; SMC, smooth muscle cells; v, venous; a, arterial; aa, arteriolar.

Fundamental understanding of vascular control is shaped by prevailing tools and the manner in which experiments are designed to probe biological behavior. In this regard, the use of vessel myography is central, as is the examination of arterial tone to agonists broadly applied to a bath. The interpretational consequence of this embedded approach is to view arterial tone as a global integrated phenomenon, among and across vessel segments, a property essential to the control of blood flow magnitude. As such, electro- and pharmaco-mechanical coupling, the latter of which incorporates receptor operated Ca^2+^ channels/Ca^2+^ sensitization, are viewed as contractile mechanisms working proportionally and synergistically with one another. Deeper examination, however, reveals a flaw in this reasoning, embodied by this query “Why are two distinct contractile mechanisms needed to regulate a singular aspect of arterial tone development and presumptively blood flow control? Indeed, integrated tone control across vessels and branch points can be achieved through electromechanical coupling alone, as charge spread through gap junctions ensures a comparable V_M_ and cytosolic Ca^2+^ response across thousands of smooth muscle cells. Could it be that pharmacomechanical coupling encodes for an aspect of blood flow delivery distinct from “magnitude”? Consider for example, a single terminal arteriole and the associated tuning of blood flow “distribution” within each capillary network. Local tuning presumptively requires focal vasomotor control, a response difficult to achieve via electromechanical coupling as the charge arise from a small number of activated cells gets readily diluted into the large mass of unstimulated cells to which they are coupled. It is in this context that the value of pharmacomechanical coupling, with its dependence on MLCP regulation and receptor operated Ca^2+^ channel activation, becomes evident. Although observations are limited, brain studies have noted that precapillary arterioles can operate in a sphincter-like manner as they branch from the penetrating arteriole. The mural cells embedded in these segments are intriguing in many aspects (Phillips et al., 2022), perhaps most notably herein, the robust expression of TRPC3 and TRPC6 (He et al., 2018; Vanlandewijck et al., 2018) (Figure 2). Vasomotor heterogeneity is also observable across the length of a penetrating arterioles, a behavior consistent although not definitive for, focal zones of enhanced TRPC channel expression and pharmacomechanical control (Institoris et al., 2015; Chow et al., 2020). What triggers focal vasomotor responses in organs like the brain, remains a question of active inquiry. Perhaps, a single astrocytic endfoot produces a diffusible signal from appropriate sensory input that tunes pharmacomechanical rather than electromechanical coupling. Arguably, pharmacomechanical regulation might also be achieved via direct neural synapses onto smooth muscle, regions of which have been recently identified by scanning electron microscopy (Zhang et al., 2024). The genesis of focal vasomotor tone should also be rationalized in a pathobiological context. Consider for example, cerebral arterial vasospasm, a profound local constriction induced by the extravasation of blood, and which elicits stroke-like symptoms. These deleterious responses are often refractory to L-type Ca^2+^ channel blockade, and thus presumptively pharmacomechanical in foundation. It follows that targeting key pathways and signaling proteins underpinning pharmacomechanical coupling may be of clinical benefit to appropriate patient populations.

## 5 Summary

A putative receptor activated TRPC channel in a cohort of smooth muscle cells, could serve as a Ca^2+^ source to drive SR Ca^2+^ wave generation and a local pharmacomechanical constrictor response, independent of membrane potential. Contrary to electromechanical coupling where an electrical signal integrated across vessel segments sets blood flow magnitude, local V_M_-independent constriction would be presumptively designed to tune distribution. In this regard, pharmacomechanical coupling should be view as functional distinctive from electromechanical coupling but essential to the optimization of demand/supply coupling to active tissues (Figure 2). While an intriguing concept, experimental validation is essential, the first step being the identification of local constrictor sites *in vivo*, a process which has begun. Further, it is interesting to consider whether such sites are molecularly unique, perhaps displaying enhanced expression of receptor operated Ca^2+^ channels. One could alternatively argue that these sites of point control receive additional inputs from surrounding parenchymal cells, for example, defined astrocytic end feet or the sparely arranged interneurons in brain. Further experimentation is clearly needed to deepen insight on this novel aspect of blood flow control. Likewise, intricate computers models of contractile control, akin to those used to probe intercellular conduction in brain microvascular networks will provide further conceptual insight and set new boundaries to our understanding of blood flow coupling to energetic demands.

## Data Availability

The original contributions presented in the study are included in the article/supplementary material, further inquiries can be directed to the corresponding author.
